# 1347. Do Class 3 Semaphorins Play a Role in COVID-19 Outcomes in Patients with Non-alcoholic Fatty Liver Disease?

**DOI:** 10.1093/ofid/ofad500.1184

**Published:** 2023-11-27

**Authors:** Martina Vargovic, Branimir Gjurasin, Lara Samadan, Adriana Vince, Neven Papic

**Affiliations:** University Hospital for Infectious Diseases Zagreb, Zagreb, Grad Zagreb, Croatia; University Hospital for Infectious Diseases Zagreb, Zagreb, Grad Zagreb, Croatia; School of Medicine, University of Zagreb, Croatia, Zagreb, Grad Zagreb, Croatia; University Hospital for Infectious Diseases Zagreb, Zagreb, Grad Zagreb, Croatia; University Hospital for Infectious Diseases Zagreb, Zagreb, Grad Zagreb, Croatia

## Abstract

**Background:**

Class 3 semaphorins are newly recognized regulators of T-lymphocyte differentiation, neutrophil migration, and the production of inflammatory cytokines, with a possible role in controlling COVID-19 “cytokine storm”. While non-alcoholic fatty liver disease (NAFLD) is associated with COVID-19 severity and outcomes, immunological mechanisms remain unclear. Since NAFLD is associated with semaphorin pathway dysregulation, this study aimed to determine the possible association of serum semaphorin concentrations with the severity of COVID-19 in patients with NAFLD.

**Methods:**

This was a secondary analysis of the prospective observational study (CovidFAT) that recruited patients with severe COVID-19 during the delta wave of the pandemic. Upon admission, patients were screened for NAFLD, routine clinical and laboratory data were collected, and serum semaphorin -3A, -3C and -3F concentrations were analyzed by ELISA. Sixty SARS-CoV2 negative, age and sex-matched healthcare workers (30 with NAFLD) were included as controls.

**Results:**

Eighty patients (44 males (55%); mean age of 58±12 years) with COVID-19 were included. Of them, 42 had NAFLD. There were no differences in age, sex, comorbidities, duration of symptoms, and disease severity at admission, except for BMI which was higher in the NAFLD group (32±8 vs 27±3 kg/m2). Patients with NAFLD had significantly higher CRP (123±70 vs 97±60 mg/L), IL-6 (92±59 vs 45±38 pg/mL), neutrophile-lymphocyte ratio (11±14 vs 6.5±2.6) and GGT (93±78 vs 54±47 IU/L). Patients with COVID-19 had significantly higher serum concentrations of SEMA3C and SEMA3F and lower of SEMA3A than healthy controls, as presented in Figure 1. Patients with COVID-19 and NAFLD had significantly higher SEMA3C and SEMA3F serum concentrations than patients without NAFLD. In ROC analysis, SEMA3A and SEMA3F showed good accuracy for differentiating moderate from severe/critical COVID-19 (AUC 0.75, CI95% 0.66-0.85 and 0.74, CI95% 0.54-0.84, respectively).

Serum semaphorin concentrations measured in healthy controls and patients with COVID-19. Shown are means with standard deviations and p-values (ANOVA test).

**Figure:**
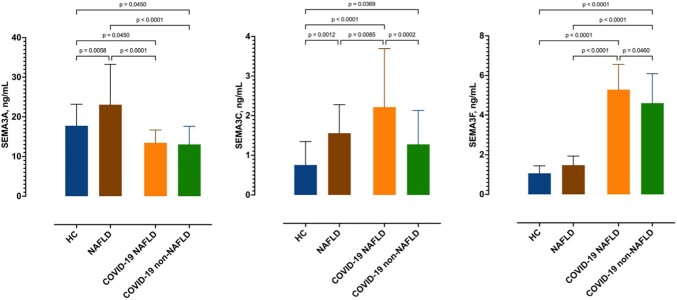

**Conclusion:**

NAFLD is associated with distinct semaphorin profiles in COVID-19, possibly associated with disease severity and adverse outcomes.

**Disclosures:**

**All Authors**: No reported disclosures

